# Emerging Approaches to Anthelmintic Therapy Using Medicinal Plants and Phytochemicals: A Review of Natural Products Against Strongyloidiasis

**DOI:** 10.3390/pathogens14090842

**Published:** 2025-08-23

**Authors:** Julio López-Abán, Belén Vicente-Santiago, Guadalupe Gutiérrez-Soto, Nancy Edith Rodríguez-Garza, Miroslava Kačániová, Iosvany López-Sandin, Cesar Iván Romo-Sáenz, Juan Manuel Ballesteros-Torres, Lucio Galaviz-Silva, Uziel Castillo-Velázquez, Stefania Garzoli, Joel Horacio Elizondo-Luévano

**Affiliations:** 1 Infectious and Tropical Diseases Group (e-INTRO), IBSAL-CIETUS (Biomedical Research Institute of Salamanca-Centre for Tropical Diseases at the University of Salamanca), Faculty of Pharmacy, Universidad de Salamanca, Ldo, Mendez Nieto s/n, 37007 Salamanca, Spain; jlaban@usal.es (J.L.-A.); belvi25@usal.es (B.V.-S.); nancy.rodriguezg@usal.es (N.E.R.-G.); 2 Laboratory of Natural Sciences, Biomolecular Innovation Group, Faculty of Agronomy, Campus of Agricultural Sciences, Universidad Autónoma de Nuevo León, Francisco I. Madero s/n, Ex Hacienda el Canada, Cd. Gral. Escobedo 66050, NL, Mexico; juanita.gutierrezst@uanl.edu.mx; 3 Department of Chemistry, Faculty of Biological Sciences, Universidad Autónoma de Nuevo León, Av. Universidad s/n, Cd. Universitaria, San Nicolás de los Garza 66455, NL, Mexico; 4 Institute of Horticulture, Faculty of Horticulture and Landscape Engineering, Slovak University of Agriculture, Tr. A. Hlinku 2, 94976 Nitra, Slovakia; miroslava.kacaniova@gmail.com; 5 School of Medical & Health Sciences, VIZJA University, Okopowa 59, 01 043 Warszawa, Poland; 6 Engineering Department, Universidad Emiliano Zapata, Avenida Rodrigo Gómez, Sector Heroico s/n, Monterrey 64260, NL, Mexico; iosvanyls@gmail.com; 7 School of Medicie and Biomedical Sciences, Universidad Autónoma de Chihuahua, Circuito Universitario, Campus II, Chihuahua 31109, CH, Mexico; cromo@uach.mx; 8 Department of Microbiology and Immunology, Faculty of Biological Sciences, Universidad Autónoma de Nuevo León, Av. Universidad s/n, Cd. Universitaria, San Nicolás de los Garza 66455, NL, Mexico; juan.ballesterostr@uanl.edu.mx; 9 Laboratory of Molecular and Experimental Pathology, Faculty of Biological Sciences, Universidad Autónoma de Nuevo León, Av. Universidad s/n, Cd. Universitaria, San Nicolás de los Garza 66455, NL, Mexico; lucio.galavizsl@uanl.edu.mx; 10 Department of Immunology, Faculty of Veterinary Medicine and Zootechny, Campus of Agricultural Sciences, Universidad Autónoma de Nuevo León, Francisco I. Madero s/n, Ex Hacienda el Canada, Cd. Gral. Escobedo 66050, NL, Mexico; uziel.castillovl@uanl.edu.mx; 11 Department of Chemistry and Technologies of Drug, Sapienza University, P. Le Aldo Moro, 5, 00185 Rome, Italy; stefania.garzoli@uniroma1.it

**Keywords:** anthelmintic activity, ethnopharmacology, helminthiasis, medicinal plants, natural products, nematodoses, strongyloidosis

## Abstract

Strongyloidosis is a parasitic disease caused by *Strongyloides stercoralis*, a nematode with a complex life cycle that facilitates long-term persistence within the host. The infection affects millions of people in tropical and subtropical regions and poses a particular challenge in immunocompromised individuals. Although conventional treatments, such as ivermectin and albendazole, are generally effective, emerging concerns regarding drug resistance and adverse effects have prompted the search for alternative therapeutic options. In this context, natural products—including plant extracts, bioactive phytochemicals, and nanoparticle-based formulations derived from natural sources—are emerging as promising anti-*Strongyloides* potential. This review summarizes recent studies on natural products with anthelmintic activity against strongyloidiasis, with emphasis on their mechanisms of action, efficacy, and future perspectives. A systematic search of the literature was conducted using terms related to *Strongyloides*, plant species, extracts, and bioactive compounds with nematocidal activity. Eligible studies included those reporting the activity of plants, plant extracts, and their purified metabolites against *Strongyloides* spp. Data were compiled into a comprehensive table including year of publication, author, plant species, active principle, application conditions, and target nematode species. The pharmacological treatment of this parasite varies according to its life cycle stage. Various biomolecules, phytoactive compounds, and novel plant-based formulations have demonstrated promising activity and may be considered both for treatment and for inclusion in control programs for strongyloidiasis. This review highlights medicinal plants and phytochemicals with ethnopharmacological background and experimentally validated activity against *Strongyloides* spp., integrating evidence from in vitro, in vivo, and experimental models, as well as clinical trials.

## 1. Introduction

Parasitic diseases remain a serious global public health problem. Intestinal parasitic nematodes are among the most widespread human parasites, infecting nearly two billion people worldwide, particularly in developing countries. Nematode infections can be broadly classified into intestinal and tissue nematodoses [1]. These diseases remain endemic in many regions, especially in tropical and subtropical areas with poor health infrastructure, where they contribute to significant morbidity. Although some infections are asymptomatic, others can cause severe illness in vulnerable individuals [2].

The most clinically important intestinal nematodoses in humans are caused by soil-transmitted helminths (STHs), roundworm (*Ascaris lumbricoides*), hookworms (*Necator americanus* and *Ancylostoma duodenale*), threadworms (*Strongyloides stercoralis*), whipworm (*Trichuris trichiura*), and pinworm (*Enterobius vermicularis*) [3]. According to the Global Burden of Disease (GBD) Study 2019, STHs affect more than one-fifth of the world’s population, producing a spectrum of outcomes ranging from asymptomatic infections to nonspecific clinical manifestations [4]. Transmission occurs through ingestion of embryonated eggs via contaminated food or hands [5], or through active penetration of the skin by third-stage larvae (L3) [6]. While *A. lumbricoides*, *T. trichiura*, and *E. vermicularis* primarily affect school-aged children, hookworms and *S. stercoralis* are more common in adults [7,8].

Strongyloidiasis, caused primarily by *S. stercoralis*, is a neglected tropical disease (NTD) that disproportionately affects populations living in poverty. Its management is complicated by the parasite’s capacity for autoinfection, as well as the limited sensitivity of current diagnostic methods [9,10]. In addition, the widespread emergence of anthelmintic resistance in veterinary nematodes has raised concerns that similar resistance may develop in human parasites, highlighting the urgent need for novel therapeutic approaches [11]. Natural products represent a promising alternative due to their structural diversity, biological activity, and relatively low toxicity [12]. Importantly, the populations most affected by these infections frequently lack access to adequate sanitation, clean water, and primary healthcare, which exacerbates transmission and reinfection cycles [13]. These infections also impair growth and cognitive development in children and contribute to iron deficiency anemia, particularly among school-aged children and pregnant women [8]. During these nematode infections, there are many molecular interactions between inflammatory cells and helminths [9].

Globally, *Strongyloides* infections are prevalent in tropical regions of Asia, Africa, and America, with prevalence decreasing farther from the equator [14]. Two main species parasitize humans: *S. stercoralis* and *S. fulleborni*. It is estimated that over 100 million individuals, including those in some temperate regions, are infected with *S. stercoralis* [15]. While infection in immunocompetent hosts is often asymptomatic, chronic disease can develop, and, in some cases, respiratory manifestations such as asthma have been attributed to this helminth [16].

Recognizing the importance of strongyloidiasis, the World Health Organization (WHO) included it alongside STHs in the 2021–2030 roadmap for NTDs [17]. Control strategies prioritize preventive chemotherapy through mass drug administration (MDA), employing albendazole in combination with ivermectin (IVM) to improve treatment efficacy [18]. However, the emergence of IVM resistance poses a serious challenge [19]. Addressing this requires genome-wide approaches to monitor resistance, development of novel drugs or formulations, validation of improved diagnostics, and strategies to overcome the complexity of multi-species STH infections. Overcoming these barriers is essential to reducing the burden of strongyloidiasis and improving global control programs [19].

Treatment of nematode infections relies primarily on antiparasitic drugs, administered either as monotherapies or in combination. These treatments are used not only for individual cases but also in control programs aimed at transmission elimination, mass treatment of high-risk groups, and post-diagnosis interventions. However, a persistent challenge is the high reinfection rate due to lifestyle and environmental factors [14]. Treatment failure is also reported, often resulting from poor adherence, sub-therapeutic dosing, counterfeit drugs, or other external factors [2]. Medicinal plants, long used in ethnomedicine for a variety of ailments [20], have gained renewed attention. Numerous recent studies demonstrate that plant extracts possess in vitro and in vivo activity against nematodes [21]. Their ethnopharmacological history, chemical diversity, and broad biological activity position medicinal plants as an attractive alternative or complementary approach to current anthelmintic drugs [22,23].

## 2. Anti-Helminthic Drugs

For practical purposes, nematodes are categorized into intestinal, filarial, and tissue species, all of which represent widespread parasitic infections globally. Infections may remain asymptomatic or cause gastrointestinal and systemic symptoms [24]. The main strategy for controlling soil-transmitted helminths (STHs) is mass drug administration (MDA) with anthelmintic drugs (Table 1). Albendazole and mebendazole remain the cornerstones of these programs. While both drugs are effective against *A. lumbricoides* [25], mebendazole shows limited efficacy against hookworm infections, and neither agent achieves satisfactory cure rates against *T. trichiura* (28% and 36%, respectively) [8,26].

*Strongyloides stercoralis* responds to benzimidazoles or ivermectin (IVM), with IVM being the treatment of choice for disseminated infections, either alone or in combination with albendazole [25]. Optimizing treatment programs requires tailoring drug selection, dosage, and frequency to local epidemiological patterns, accounting for variations in STH prevalence and species distribution. Mathematical modeling and epidemiological studies are increasingly used to guide treatment strategies and to design more effective control programs [8].

### 2.1. Effect of Pharmacological Treatments on Strongyloides spp.

Various pharmacological strategies have been evaluated as therapeutic alternatives against *Strongyloides* infections. Among them, commonly used drugs such as albendazole, ivermectin, and mebendazole have demonstrated efficacy, both in reducing parasite burden and inhibiting oviposition.

According to Clarke et al. (2019), drug combinations significantly enhance clinical effectiveness, measured through parameters such as relative risk (RR) of cure and the difference in egg reduction rate (dERR) [28]. For instance, the albendazole–ivermectin combination showed an RR of 3.22, while albendazole–oxantel pamoate reached an RR of 5.7. Other combinations, such as mebendazole–ivermectin and tribendimidine–oxantel pamoate, reported values of 3.37 and 4.06, respectively. Regarding dERR, albendazole–ivermectin and albendazole–oxantel pamoate achieved values of 0.97 and 0.51, indicating high efficacy in reducing variability in egg output. These findings support the use of combination therapies as an effective alternative to enhance treatment and control of *Strongyloides* infections.

Other promising alternatives under investigation include the development of triazole derivatives, originally designed as antifungal agents. Through structural modifications or conjugation with organometallic compounds, these molecules have shown the ability to interfere with nematode nervous system receptors and metabolic enzymes, suggesting potential future application as anti-*Strongyloides* treatments [29].

Additionally, bacterial-derived molecules such as Cry crystal toxins from *Bacillus thuringiensis* have been studied. Although most evidence comes from non-parasitic nematode models, their ability to disrupt intestinal membrane integrity and induce neuronal degeneration indicates a potential role as candidates for anti-*Strongyloides* therapies [30].

Taken together, these findings highlight that both synthetic drugs used in strategic combinations and compounds derived from novel platforms (modified triazoles and bacterial toxins) represent promising avenues for developing more effective therapies with innovative mechanisms of action against *Strongyloides* spp.

### 2.2. Drug Targets

The identification and validation of suitable biological targets represent crucial steps in the discovery and development of novel anthelmintic drugs [31]. Target validation for specific clinical applications is feasible only when commercially approved drugs exist with a well-established mechanism of action against the same target [32]. However, this poses a significant challenge in soil-transmitted helminths (STHs), since the precise molecular targets of many currently used anthelmintics remain poorly characterized [33]. For *Strongyloides* spp., potential targets under investigation include neuromuscular ion channels (e.g., glutamate-gated chloride channels and nicotinic acetylcholine receptors), energy metabolism enzymes (e.g., fumarate reductase and mitochondrial electron transport proteins), and cytoskeletal proteins such as tubulin. Each of these targets offers distinct opportunities for the rational design of compounds with improved specificity and potency.

### 2.3. Resistance to Anti-Helminthic Drugs

Anthelmintic drugs, widely used to treat parasitic worms, are traditionally classified according to the type of parasite they target: nematodes (roundworms), trematodes (flukes), and cestodes (tapeworms) [34]. Their primary goal is to eliminate or incapacitate worms while minimizing harm to the host. However, the discovery and development of new anthelmintics are hampered by limited financial incentives and the relatively small global market for antiparasitic therapeutics [35].

Resistance to anthelmintics has become a growing concern, particularly in veterinary medicine, where it is widespread in livestock nematodes and threatens to extend to human intestinal helminths [34]. Resistance mechanisms may include alterations in drug targets (e.g., mutations in β-tubulin associated with benzimidazole resistance), enhanced drug efflux via ABC transporters, increased detoxification through metabolic enzymes, or changes in drug absorption and sequestration within the parasite [36].

Management strategies can help mitigate resistance development [37]. These include identifying novel drug targets, designing drugs with alternative mechanisms of action, employing rotational use of different drug classes, and maintaining refugia by leaving a proportion of the parasite population untreated, thereby reducing selective pressure [34,38]. Such integrated approaches are increasingly recognized as essential to preserving the long-term efficacy of available treatments and to delaying resistance in both animal and human populations [39]. Table 2 summarizes the major mechanisms of resistance targets that have been proposed for the treatment of *Strongyloides* infections.

## 3. Pharmaceutical Uses of Natural Products

The use of natural products from medicinal plants as anthelmintic agents carries important pharmaceutical implications. These compounds represent potential sources for the discovery and development of new anthelminthic drugs [44]. The isolation, identification, and characterization of bioactive molecules from medicinal plants provide valuable opportunities for drug discovery pipelines [45,46]. Natural products display diverse chemical structures and unique mechanisms of action, offering a promising means to overcome the challenge of drug resistance commonly observed with conventional anthelmintic therapies.

Moreover, the growing global interest in plant-based medicines is supported by their potential advantages, including fewer side effects [47,48] and reduced environmental impact compared to synthetic drugs [49]. Thus, natural products not only represent a therapeutic alternative but also align with sustainable and patient-friendly healthcare strategies.

The exploration of medicinal plants against helminthiases opens new avenues for pharmaceutical research and development [50,51]. However, several challenges remain [5]. The identification and isolation of active compounds require extensive phytochemical and pharmacological investigations. Standardization of extraction procedures, formulation development, and quality control are critical to ensure reproducible safety and efficacy in antiparasitic drug development [52]. Furthermore, systematic studies on safety, pharmacokinetics, and clinical efficacy are essential to validate therapeutic potential and to support the integration of these natural compounds into modern healthcare systems [53].

## 4. Methodology

### 4.1. Research Question

This study evaluates the efficacy of medicinal plants, and their extracts, phytochemicals, and derivatives—either in their native form or incorporated into nanomaterial-based delivery systems—against *Strongyloides* species in experimental models.

We integrated evidence from both in vitro and in vivo studies to assess not only the antiparasitic potential of these natural products but also their possible molecular targets.

### 4.2. Study Design and Selection Criteria

We conducted a search of the literature for studies published between 1976 and 2025 investigating the anthelmintic activity of natural products, extracts, and plant-derived compounds against various *Strongyloides* species. The main databases consulted were Google Scholar, Scopus, Web of Science, ResearchGate, Latindex, and PubMed, with searches performed up to 21 July 2025.

The variables analyzed in this review included the following: plant family, plant part used, extraction method, isolated compound (s), inhibitory effects on parasites, and the type of experimental model (in vitro, in vivo, or in silico) (Table 3).

The search strategy incorporated Medical Subject Headings (MeSH) terms, including “Soil-transmitted helminths” and “*Strongyloides*”. Table 3 and Table 4 were prepared to systematically compile the qualitative and quantitative data from the selected articles.

Data were extracted by author, year of publication, and study design. Studies that did not meet the inclusion criteria were excluded. The selection process involved an initial screening of titles and abstracts, followed by a full-text review. Articles were independently evaluated by three reviewers (J.L.-A., N.E.R.-G., and J.H.E.-L.). Any discrepancies were resolved through discussion with other members of the study team (J.M.B.-T., M.K., C.I.R.-S., S.G., and L.G.-S.).

## 5. Results

### 5.1. Medicinal Plants with Anti-Strongyloides Activity

Several medicinal plants and natural products have demonstrated promising activity against nematodes, including *Strongyloides* spp., and may represent viable alternatives or adjuncts to conventional treatments [52]. These natural products often contain bioactive compounds with anthelmintic properties capable of disrupting the parasite life cycle or survival [54,55].

One notable example is the Mexican poppy (*Argemone mexicana*), whose major alkaloids—such as berberine and protopine—have shown activity against various geohelminths as *Strongyloides venezuelensis* [56], as well as the trematode *Schistosoma mansoni* and mosquito larvae (*Culex quinquefasciatus* and *Aedes aegypti*) [21,57,58,59]. Interestingly, protopine has demonstrated anti-HIV activity [60].

Turmeric (*Curcuma longa*), whose active component curcumin possesses strong antiparasitic properties, has also been shown effective against *S. venezuelensis* [61]. *Carica papaya* seed latex, rich in papain, has exhibited significant in vitro ovicidal and larvicidal effects against *S. venezuelensis* eggs and third-stage (L3) larvae [62]. Similarly, *Azadirachta indica* (neem tree) contains bioactive compounds such as azadirachtin, which display broad antiparasitic activity against helminths, including *Strongyloides* spp. [63]. Garlic (*Allium sativum*), due to its allicin content, has demonstrated both in vitro and in vivo anthelmintic activity against gastrointestinal helminths [64]. 

Other medicinal plants—such as *Artemisia absinthium*, *Punica granatum*, *Nigella sativa*, and *Terminalia chebula*—have shown efficacy in larval motility inhibition assays. In many cases, structural damage to larvae has been confirmed through electron microscopy [63].

*Strongyloides* nematode infections are prevalent across tropical areas of Asia, Africa, and the Americas [65,66,67,68], where communities often rely on traditional medicine due to limited healthcare access. Empirical use of medicinal plants for anti-parasitic purposes is deeply rooted in these regions.

While the therapeutic potential of these natural products is evident, further research is required to determine their efficacy, appropriate dosages, and safety profiles in the treatment of STH infections and strongyloidiasis. Importantly, these natural remedies should be investigated as complementary strategies and administered under proper medical supervision alongside standard therapies for parasitic infections. Table 5 provides a compilation of plants documented to have activity against *Strongyloides* spp.

### 5.2. Medicinal Biomolecules with Anti-Strongyloides Activity

Medicinal biomolecules with anti-*Strongyloides* activity are a promising area of study. These natural products often contain bioactive compounds with anthelmintic properties, which can disrupt the parasites’ life cycle or survival.

Beyond crude extracts, various purified plant-derived compounds have been investigated for their efficacy against intestinal nematodes [129]. These studies have utilized experimental platforms including in vitro bioassays, in vivo models, and computational simulations.

Specifically, pure phytochemicals—including alkaloids, flavonoids, tannins, and proteolytic enzymes—have been evaluated for their antiparasitic effects using in vitro, in vivo, and in silico models (Table 6) [63].

Berberine, an isoquinoline alkaloid derived from *Argemone mexicana*, has demonstrated potent in vitro larvicidal activity against *S. venezuelensis* third-stage larvae (L3) [109]. Similarly, the polyphenols quercetin and curcumin have shown significant inhibitory effects on *S. venezuelensis* L3 motility and survival [21]. Papain, a cysteine protease extracted from the seed latex of *Carica papaya*, exhibited strong in vitro inhibition of egg hatching and larval motility against *S. venezuelensis* [62]. Thymoquinone, the main bioactive compound of *Nigella sativa* seeds, produced in vitro nematicidal effects, with in silico molecular modeling indicating a possible mechanism through the inhibition of mitochondrial enzymes [63].

Condensed tannins from *Punica granatum* and *Acacia nilotica* have been reported to disrupt larval cuticle proteins, resulting in immobility and death, as confirmed by both microscopy and proteomic analyses [63]. Moreover, several phytochemicals—including berberine and quercetin—exhibit favorable molecular docking scores at the colchicine-binding site of α- or β-tubulin, suggesting that microtubule destabilization could be an additional mechanism underlying their antiparasitic activity (Table 7).

These findings highlight the potential of plant-derived molecules to serve as scaffolds for novel anthelmintic agents. Furthermore, their integration into combination therapies with existing drugs may enhance efficacy, broaden the spectrum of activity, and mitigate the development of drug resistance in the treatment of *Strongyloides* infections.

### 5.3. Anti-Strongyloides Nanoparticles

The application of nanomaterials in the formulation of natural compounds has significantly enhanced their bioavailability, stability, and efficacy against parasitic infection parasites [61,143,144]. Several nanoparticle-based strategies have been investigated. For instance, poly(lactic-co-glycolic acid) (PLGA) nanoparticles enable the controlled release and targeted delivery of bioactive molecules, thereby prolonging therapeutic effects and reducing dosing frequency [145,146]. Silver nanoparticles exhibit intrinsic anthelmintic activity and have been shown to act synergistically with plant-derived extracts, enhancing overall antiparasitic efficacy [147,148]. Synthetic polymer-based nanoparticles, such as those formulated with Eudragit^®^ (^©^Evonik Industries AG, Essen, Germany), can improve the intestinal absorption of metabolites with low aqueous solubility, potentially increasing systemic availability and therapeutic potency [149,150,151].

In addition to these systems, various nanoparticle formulations incorporating ivermectin (IVM) have been explored for their antiparasitic and anthelmintic effects [152]. Notably, a poly(ε-caprolactone) (PCL)-based nanoparticle formulation loaded with IVM demonstrated superior activity against adult female *Strongyloides venezuelensis* compared with non-encapsulated IVM, suggesting that nanoencapsulation can enhance drug–parasite interactions and efficacy [125,153]. Furthermore, inorganic nanoparticles, including iron oxide, zinc oxide, and aluminum oxide, have exhibited nematocidal effects against *Strongyloides* eggs [154]. Beyond *Strongyloides*, nanoparticle-based approaches have shown activity against other soil-transmitted helminths (STHs) such as *H. contortus*, *Pheretima posthuma*, and *Toxocara vitulorum*, among others [155].

These findings indicate that nanoparticle systems—particularly when combined with bioactive natural products—hold considerable promise as alternative or complementary strategies for the management of animal and human nematodoses. This remains a broad and promising field of research, with significant potential for the development of novel formulations targeting STHs and other parasitic nematodes [155].

## 6. Limitations

Despite the promising findings presented in this review regarding the potential of natural products and plant-derived compounds against *Strongyloides* infections, several limitations must be acknowledged. First, the inherent risks and biases of in vitro and in vivo studies may affect the generalizability of results to clinical practice. Many of the reported activities rely on preliminary assays, often conducted with crude extracts or partially characterized fractions, which may not fully represent the efficacy and safety of the active principles in humans.

Another important consideration is the possible adverse effects of the extracts. Although plants have been widely used in traditional medicine, the safety profile of many species remains insufficiently documented, and some phytochemicals may exert cytotoxic, immunomodulatory, or pro-oxidant effects at higher concentrations. Additionally, potential interactions with conventional medications pose a challenge, particularly in endemic areas where populations often combine herbal remedies with prescribed antiparasitic drugs. Such interactions could compromise efficacy or increase the risk of toxicity [28,156,157].

From a translational perspective, there are also significant obstacles to standardization, patenting, approval, and commercialization of herbal-derived products [158]. The chemical composition of extracts is highly dependent on factors such as plant origin, harvesting season, and extraction methodology, which complicates reproducibility [159]. Furthermore, the lack of standardized protocols for quality control hinders regulatory approval. Intellectual property issues surrounding traditional knowledge also raise ethical and legal challenges, particularly for patenting and commercialization.

Overall, plant-based therapies represent a valuable complementary strategy for the control of nematode infections [63,160]. Overcoming these limitations will require rigorous preclinical and clinical validation, integration of pharmacological and toxicological studies, and the development of harmonized regulatory frameworks [158].

## 7. Discussion and Conclusions

Medicinal plants and their derivatives remain a valuable resource in the development of novel anthelmintic therapies [158,159]. Natural products represent a sustainable and cost-effective alternative for managing strongyloidiasis [63]. The evidence compiled in this review highlights the significant potential of plant extracts, purified phytochemicals, and advanced formulations—particularly, nanoparticle-based systems—in combating *Strongyloides* infections. These natural agents have demonstrated efficacy in various in vitro, in vivo, and in silico models, with mechanisms of action ranging from interference with parasite metabolism to the inhibition of specific molecular targets [55,63,161].

The global burden of *Strongyloides* infection remains a major public health challenge, particularly in tropical and subtropical regions of Asia, Africa, and Latin America, where poor sanitation and limited access to medical care contribute to its persistence [4,162,163,164]. Conventional chemotherapeutics such as ivermectin and albendazole have been widely used with relative success [165,166]; however, emerging reports of reduced efficacy, potential resistance, and adverse side effects highlight the urgent need for alternative or complementary therapies.

Medicinal plants and phytochemicals have historically been employed in endemic areas as empirical remedies against intestinal helminthiases, including strongyloidiasis. Ethnobotanical surveys from communities in Brazil, Mexico, India, and sub-Saharan Africa demonstrate that populations at risk often rely on preparations derived from *Chenopodium ambrosioides*, *Carica papaya*, *Azadirachta indica*, and *Artemisia* species, among others, as anthelmintic agents, as shown in Table 5 and Table 6. These traditional practices not only provide valuable leads for pharmacological research but also emphasize the role of cultural knowledge in managing neglected parasitic diseases.

The evidence compiled in this review underscores the potential of phytochemicals such as alkaloids, flavonoids, terpenoids, and saponins to exert direct nematocidal activity or to interfere with parasite survival through oxidative stress modulation, inhibition of neuromuscular function, or disruption of cuticular integrity. Moreover, nano- and microencapsulation strategies using biocompatible polymers like PLGA are opening new possibilities to enhance the bioavailability, targeted delivery [152,153], and stability of plant-derived compounds [167], addressing a major limitation in the translation of natural products into clinical use.

Nevertheless, most studies remain confined to in vitro assays or preliminary in vivo experiments in animal models, and robust clinical trials are still lacking. The gap between traditional knowledge and modern pharmacological validation highlights the importance of integrating ethnopharmacology with contemporary methodologies such as high-throughput screening, metabolomics, and molecular docking. These approaches can accelerate the identification of bioactive compounds and elucidate their mechanisms of action at the host–parasite interface.

In conclusion, medicinal plants and their phytochemicals represent promising candidates for the development of novel anthelmintic therapies against strongyloidiasis. Future efforts should focus on rigorous preclinical and clinical studies, the application of advanced formulation technologies, and the conservation of ethnobotanical knowledge. By bridging traditional practices with modern scientific strategies, natural products may provide a sustainable and effective solution to control this neglected tropical disease.

Future research should focus on optimizing extraction techniques, standardizing quality control, and conducting well-designed preclinical and clinical studies to validate their efficacy and safety. Additionally, exploring synergistic effects with existing anthelmintics and combining them with immunomodulatory agents could enhance therapeutic outcomes while reducing the likelihood of resistance. The sustainable development of plant-based interventions offers an accessible, cost-effective, and innovative pathway toward improved management of strongyloidiasis.

## Figures and Tables

**Table 1:** Anthelmintic drugs used against *Strongyloides*.

Drug	Activity	Reference
Albendazole	++	[1,24,26,27]
Ivermectin	+++	
Mebendazole	+	
Pyrantel pamoate	−	

−: Negative activity; +: Good activity; ++: High activity; +++: Very high activity.

**Table 2:** Anthelmintic mechanisms of resistance.

Anthelmintic Drug	Year		Mechanism	Reference
	Initial Approval	First Report of Resistance Published		
Benzimidazoles	1961	1964	Mutations: β-tubulin; Phe200Try, Phe167Try or Glu198Ala.	[40,41,42,43]
Imidothiazoles-tetrahydropyrimidines	1970	1979	Changes in nicotinic acetylcholine receptors.	
Avermectin-mylbemicins	1981	1988	Sensitivity reducted of GluCl/GABA receptors.	

Ala: Alanine; Phe: Phenylalanine; Try: Tryptophan; Glu: Glutamate; GluCl: Glutamate-gated chloride channels; and GABA: gamma-aminobutyric acid.

**Table 3:** Extraction of data from bibliographic references to establish the status of treatment against nematodes that affect humans.

Variable	Study Cases
General subjects	Efficacy, safety, resistance, and natural products.
Year of publication	From 1 January 1976 to 21 July 2025.
Pharmaceutical or natural product	Name of the phytopharmaceutical, extract, molecule/metabolite.
Conditions of application	Base product, combinations and formulations (oral route, tablets, macroparticles, complementary treatment, alternative treatment, application patterns, model, topical, and subcutaneous).
Parasite	*Strongyloides*.
Phase	Egg; larvae.
Population	Children, Adults, General population.
Type of study	Experimental model (in vitro, in vivo, and in silico), original research, review, systematic review, and clinical trial.

**Table 4:** Search strategy.

Search Strategy						
Concept 1		Concept 2		Concept 3		Concept 4
*Strongyloides*	AND	Strongyloidosis	AND	Natural product,	AND	In vivo
				Plant,		OR
				Extract OR Extraction,		In vitro
				Purification,		OR
				Isolation		In silico

Boolean operators: AND, OR.

**Table 5:** Potential plants with anti-*Strongyloides*’s activity.

Plant Species	*Strongyloides* Specie	Formulation	Model	Activity	Authors, Published Year [Reference]
*Jatropha curcas*	*Strongyloides* infections	Not specified.	In vivo	Preliminary observations on the anti-*Strongyloides* activity in goats and sheep.	Adam & Magzoub, 1976 [69]
*Ficus glabrata*	S.s.	Latex	In vivo	Reduction in egg and larvae output by 42%. Evaluation in a location in Peru. 1 cm^3^/kg per day for three consecutive days.	Hansson et al., 1986 [70]
*Pyrethrum marc*	*Strongyloides* spp.	Dorper sheep were fed a diet based on the *P. marc* plant.	In vivo	Fecal egg counts (FEC) reduction in sheep. Treatments: 36 mg/kg body weight at days 0, 2, 4, 6, 8, and 10. The larvae recovered were 0.8%.	Mbaria et al., 1998 [71]
*Khaya* *senegalensis*	*Strongyloides* sp.	Aq. and EtOH extracts.	In vitro and In vivo	Anthelmintic activity against gastrointestinal nematodes of sheep. In vitro test (LC_50_ or LD_50_ in mg/mL at 7 days): LC_50_ of aqueous extract: 0.7. LD_50_ of ethanolic extract: 0.5. In vivo test (FEC %): 125 mg/Kg: 33% FEC at day 3 to 9. 250 mg/Kg: 75% FEC at day 9 to 12. 500 mg/Kg: 0% FEC at day 12.	Ademola et al., 2004 [72]
*Artemisia brevifolia*	S.p.	In vitro assays: Crude Aq. and MeOH extracts. In vivo assays: The crude powder, Crude Aq. and MeOH extracts.	In vivo and In vitro	Dose-dependent anthelmintic activity	Iqbal et al., 2004 [73]
*Cardiospermum halicacabum*	S.s.	Lyophilized Aq. and alcohol extracts.	In vitro	In vitro effect of *C. halicacabum* extracts for their effectiveness against L3 of S.s.	Boonmars et al., 2005 [74]
*Spondias mombin*	*Strongyloides* spp.	Aq. and EtOH Extracts.	In vitro and In vivo	Anthelmintic activity against gastrointestinal nematodes (L3) of sheep. In vitro test LC_50_ in mg/mL: Aqueous extract = 1; Ethanolic extract = 0.5. In vivo test (FEC %): 0.125 g/Kg: 10% reduction in FEC at day 3 to 12. 0.25 g/Kg: 60% reduction in FEC at day 3 to 12. 0.5 g/Kg: 65% reduction of FEC at day 3 to 12.	Ademola et al., 2005 [75]
*Nicotiana tabacum*	S.p.	Crude Aq. and MeOH extract.	In vivo and In vitro	Aq. and MeOH extracts of *N. tabacum* exhibit dose-dependent anthelmintic activity both in vitro and in vivo.	Iqbal et al., 2006 [76]
*Nauclea latifolia*	*Strongyloides* spp.	Aq. and EtOH extracts.	In vitro and In vivo	Anthelmintic activity against gastrointestinal nematodes (L3) of sheep. In vitro test LC_50_ in mg/mL: Aqueous extract = 0.7; Ethanolic extract = 0.7. In vivo test (FEC %): 0.25 g/Kg: 30% reduction in FEC at day 3 to 12. 0.5 g/Kg: 60% reduction of FEC at day 3 to 12.	Ademola et al., 2007b [77]
*Spigelia anthelmia*	*Strongyloides* sp.	Aq. and EtOH extracts.	In vitro and In vivo	Anthelmintic activity against gastrointestinal nematodes of sheep. In vitro test LC_50_ in mg/mL: Aqueous extract = 0.71; Ethanolic extract = 0.63. In vivo test (FEC %): 0.125 g/Kg: 23% reduction in FEC at day 3. 0.25 g/Kg: 74% reduction in FEC at day 3. 0.5 g/Kg: 75% reduction of FEC at day 3.	Ademola et al., 2007a [78]
*Acacia negra* (*Acacia mearnsii*)	S.p.	Tannin from *A. negra*	In vivo	A diet containing condensed tannins from the *A. negra* decreased the presence of S.p. eggs and larvae in feces of infected lambs.	Cenci et al., 2007 [79]
*Carica papaya*	S.s.	Air-dried *C. papaya* seeds.	In vivo	LM examination of wet preparations of freshly passed stools to confirm the presence of intestinal parasites, their larvae, and ova. 100% effectiveness after 7 days.	Okeniyi et al., 2007 [80]
*Fusarium parviflora*	S.p. and other gastrointestinal nematodes	Aq. and EtOH extracts.	In vivo and In vitro	Effect in hatch and LDI in in vitro and FEC reduction in in vivo tests.	Al-Shaibani et al., 2009 [81]
20 indigenous medicinal plants of Bangladesh	*Strongyloides* sp.	Aq. extracts	In vivo	Dose-dependent anthelmintic activity.	Amin et al., 2010 [82]
*Carapa guianensis*	*Strongyloides* sp.	Seed oil	In vitro	Ovicidal and larvicidal effect against gastrointestinal nematodes of goats and sheep.	Farias et al., 2010 [83]
*Azadirachta indica*	S.p. and other gastrointestinal nematodes	Crude powder, crude Aq. and MeOH extracts of *A. indica* seeds.	In vivo	Dose-dependent anthelmintic activity. FEC and larval reduction and counts post-treatments from coprocultures of sheep.	Iqbal et al., 2010 [84]
*Elephantorrhiza elephantina*	*Strongyloides* sp.	MeOH extract, *n*-hexane, EtOAc and Aq. fractions.	In vitro and In vivo	Reduction % of FEC and larval count. Effect on body weight of sheep.	Maphosa & Masika, 2012 [85]
*Piper tuberculatum* and *Lippia sidoides*	S.v.	Essential oil of *L*. *sidoides* and extract of *P. tuberculatum*.	In vivo	Effect in *Rattus norvegicus*: Decrease in the presence of adult worms recovered from the initial third of the intestine of rats.	Carvalho et al., 2012 [86]
*Zanthoxylum zanthoxyloides* and *Newbouldia laevis*	S.r.	Hydrodistillation and essential oils.	In vitro	Essential oil activity of *Z. zanthoxyloides*: Egg hatching IC_50_ and IC_90_ in μg/mL: IC_50_ = 18; IC_90_ = 29. Larval migration IC_50_ and IC_90_ in μg/mL: IC_50_ = 47; IC_90_ = 165. Essential oil activity of *N. laevis*: Egg hatching IC_50_ and IC_90_ in μg/mL: IC_50_ = 20; IC_90_ = 38. Larval migration IC_50_ and IC_90_ in μg/mL: IC_50_ = 52; IC_90_ = 146.	Olounladé et al., 2012 [87]
*Mangifera indica*	S.s.	Aq. extracts of immature fruits.	In vitro	100% inhibition of LDI (L3) at 100 mg/mL^−1^ (6 h post treatment).	El-sherbini & Osman, 2013 [88]
*Anogeissus leiocarpus*	S.p.	EtOH extract.	In vitro	The administration of 80 mg/kg EtOH extract (single oral dose) induced high efficacy (100%) against adult S.p.	Soro et al., 2013 [89]
*Eryngium foetidum*	S.s.	MeOH-water (4:1, *v*/*v*) crude extracts and its main compound trans-2-dodecenal (eryngial).	In vitro	Anthelmintic activity against infective third-stage larvae (L3) of S.s.	Forbes et al., 2014 [90]
*Fusarium parviflora*	S.p.	Aq. and EtOH extracts of *F. parviflora*.	In vivo and In vitro	*F. parviflora*: Effect in LDI and FEC.	Bauri et al., 2015 [91]
*Eucalyptus globulus*	S.p.	Crude Aq. and MeOH extract.	In vivo and In vitro	Efficacy of extracts on ovine gastrointestinal nematodes. In vitro test ED_50_ and ED_99_ in mg/mL: Aqueous extract: EHT: ED_50_ = 1.5; ED_99_ = 7.1 LDT: ED_50_ = 20; ED_99_ = 109 MeOH extract: EHT: ED_50_ = 3.76; ED_99_ = 33.81 LDT: ED_50_ = 15.6; ED_99_ = 94.5 In vivo test FEC %: Aqueous extract: LDT = 5 g/single oral dose. 66% of FEC reduction was observed in in vivo test on day 21 post-treatment, although in initial stages it showed 58.0 and 80% effectiveness on days 7 and 14 post-treatment.	Kanojiya et al., 2015 [92]
*Albizia lophantha*	S.w.	Alcoholic extract.	In vitro	Dose-dependent anthelmintic (reduction in LDI and FEC) activity.	Chicaiza-Tisalema et al., 2016 [93]
*Lawsonia inermis*	S.s. and *Strongyloides* spp.	MeOH extract and *n*-hexane subpartition.	In vitro	SEM determination of ultrastructural changes in L3 caused by extracts after 24, 48, 72 and 96 h of incubation.	Ismail et al., 2016 [94]
*Lotus corniculatus*	S.p.	EtOH extracts.	In vitro	Inhibition of egg hatching S.p.	Rodríguez-Molano, C. E., Cely-Reatiga, Y. and Gómez-Lara, D. F., 2016 [95]
*Ruta graveolens*	S.s.	Boiled infusion, taken as tea three times a day for 6 to 7 days.	In situ	It mentions that the ethereal extract of the leaves has been shown to have anthelmintic activity against S.s., *Artcylostoma caninum*, and *A. duodenale*, and the essential oil against *Ascaris suilla*, *Hirudo medicinalis*, *Tubifex riuolorum*, and *Anguillula aceti*.	Malik et al., 2016 [96]
24 plant species from the following families: *Anacardiaceae*, *Arecaceae*, *Celastraceae*, *Fabaceae*, *Jungladaceae*, *Malpighiaceae*, *Myrtaceae*, *Proteaceae*, *Sapindaceae*, *Sapotaceae*, *Tiliaceae*, and *Urticaceae*	S.v.	EtOH extracts and fractions.	In vitro	The correlation between time, motility, oviposition of S.v., and mortality with the concentration of each of the extracts was determined after 72 h.	Bastos et al., 2017 [97]
*Spondias mombin*	S.v.	EtOH extracts and fractions.	In vitro	*S. mombin* EtOH extract and aqueous fraction showed 100% mortality rate after 72 h, for all tested concentrations.	Bastos et al., 2017 [97]
*C. papaya*	S.v.	Lyophilized, fresh and frozen latex; and purified papain.	In vitro	In vitro efficacy of latex and papain against S.v. larvae and FEC after 48 h.	Moraes et al., 2017 [62]
*Momordica charantia*	*Strongyloides* spp.	Aq. extract of leaves and seeds.	In vivo	Aq. extract of M. charantia leaves and seeds showed efficacy against *Strongyloides* sp. in the cattle.	Poolperm & Jiraungkoorskul, 2017 [98]
Many plants	S.s. and other filarial nematodes and some STHs.	A variety of plant extracts.	In vivo and In vitro	A variety of anthelmintic properties.	Romero-Benavides et al., 2017 [99]
*Cardiospermum halicacabum*	S.s.	Aq. and alcohol extracts.	In vitro	Showed reduction in the viability of L3.	Sunita et al., 2017 [100]
*Piper retrofractum*, *Abelmoschus esculentus*, and *C. papaya*	S.s.	EtOH extracts.	In vitro	LC_50_ in mg/mL at 24 h: *P. retrofractum* = 0.04; *A. esculentus* = 0.09; *C. papaya* = 0.10. LC_99_ in mg/mL at 24 h: *P. retrofractum* = 0.13; *A. esculentus* = 0.60; *C. papaya* = 0.48.	Sangkhantree et al., 2018 [101]
*C. papaya*	S.v.	Seed hexane extract.	In vitro	Ovicidal and larvicidal (L3) activity.	Cabral et al., 2019 [102]
*Siparuna guianensis*	S.v.	EtOH extract, ethyl acetate and Aq. fractions, essential oil and α-bisabolol.	In vitro	Anthelmintic properties of extracts and isolated compounds S.v. eggs (after 48 h) and L3 (after 24 h).	Carvalho et al., 2019 [103]
*Cassia occidentalis* and *Euphorbia hirta*	*Strongyloides* spp.	Leaf hydroalcoholic extract.	In vivo and In vitro	Both plant extracts disrupt lifecycles by suppressing the egg-laying capacity in adult worms and killing infective larvae.	Nsereko et al., 2019 [104]
*Leucaena leucocephala*	*Strongyloides* spp. and other gastrointestinal nematodes	Hydroalcoholic extract.	In vivo and In vitro	The hydroalcoholic extract pods act by inhibiting FEC and viability of L3.	Rivero-Perez et al., 2019 [105]
*Allium sativum*	Gastrointestinal nematodes	Garlic powder.	In vivo	Effects on growth performance, Rumen fermentation, and the health of lambs infected by gastrointestinal nematodes.	Zhong et al., 2019 [106]
Aqueous tinctures of 48 plant species from the following families: *Anacardiaceae*, *Araliaceae*, *Asparagaceae*, *Berberidaceae*, *Bignoniaceae*, *Calycanthaceae*, *Celastraceae*, *Colchicaceae*, *Cupressaceae*, *Dennstaedtiaceae*, *Eucommiaceae*, *Fabaceae*, *Fagaceae*, *Geraniaceae*, *Ginkgoaceae*, *Lamiaceae*, *Magnoliaceae*, *Moraceae*, *Phyllanthaceae*, *Pinaceae*, *Ranunculaceae*, *Rosaceae*, *Rutaceae*, *Simaroubaceae*, *Tamaricaceae*, *Taxaceae*, and *Vitaceae*	L3 of S.p. and *Haemonchus contortus*	Aqueous tincture.	In vitro	Dose-dependent anthelmintic activity.	Boyko et al., 2020 [107]
*Piper retrofractum*	S.s.	Hexane extract.	In vitro	Nematocidal effect on morphology and ultrastructure of S.s. L3. The L3 were evaluated for structural alterations by LM, SEM, and TEM.	Riyong et al., 2020 [108]
*Argemone mexicana*	S.v.	MeOH extract and sub-partition.	In vitro	LC_50_ in μg/mL at 96 h: Methanolic extract = 92.1. Methanol subfraction = 19.5.	Elizondo-Luévano et al., 2021a [109]
*Manilkara zapota*	S.v.	EtOH extract, fractions, and isolated compounds (Chlorogenic acid and myricitrin).	In vitro	Larvicidal (L4) activity.	Mourão Mulvaney et al., 2021 [110]
80 plant species from the following families: *Amaryllidoideae*, *Apiaceae*, *Apocynaceae*, *Aristolochiaceae*, *Asparagaceae*, *Asteraceae*, *Convolvulaceae*, *Cornaceae*, *Dipsacaceae*, *Ericaceae*, *Euphorbiaceae*, *Fabaceae*, *Hypericaceae*, *Lamiaceae*, *Lamiaceae*, *Linaceae*, *Malvaceae*, *Oleaceae*, *Onagraceae*, *Poaceae*, *Polygonaceae*, *Ranunculaceae*, *Resedaceae*, *Rosaceae*, *Sapindaceae*, *Solanaceae*, *Typhaceae*, *Ulmaceae*, *Urticaceae* and *Violaceae*	S.p.	Aq. tinctures.	In vitro	Mortality % of rhabditiform larvae (L1–2) of S.p. exposed to aqueous tinctures of leaves from 80 species of plants for 24 h.	Boyko & Brygadyrenko, 2021 [111]
*Zingiber officinale* (Ginger)	S.rn.	Ginger powder.	In vivo	The ginger powder was effective in reducing egg shed and keeping the parasitic load of S.rn. and strongyle eggs constantly low for six weeks after treatment.	Kiambom et al., 2021 [112]
*Glycyrrhiza glabra*	S.p. and other gastrointestinal nematodes	Root Aq. extract and glycyrrhetinic acid.	In vivo and In vitro	Dose-dependent anthelmintic activity, and FEC and LDI reduction. Inhibition of larval migration.	Maestrini et al., 2021 [113]
*Ginkgo biloba*	S.p.	Aq. extract.	In vitro	92.3% mortality rate of nematode larvae at 3% of plant extract solution.	Adak & Kumar, 2022 [114]
Herbal anthelmintic agents	S.p., S.s. and other nematodes	Diverse natural compounds.	In vivo and In vitro	A review of diverse anthelmintic properties.	Adak & Kumar, 2022 [114]
*Campomanesia* *xanthocarpa*	S.s.	EtOH extracts.	In vitro	Demonstrated delayed in FEC, but no anthelmintic activity against S.s.	Burgos Cantoni & Rodríguez, 2022 [115]
*Albatrellus* *confluens*	S.r.	MeOH extract; *n*-hexane, chloroform, EtOAc fractions; subfractions; and synthesized compounds.	In vitro	Larvicidal (L3) % activity.	Dube, Llanes et al., 2022 [116]
*Moringa oleifera*	S.r.	Crude MeOH extracts.	In vitro	*M. oleifera* leaves MeOH extract exhibited the 58.2% activity against S.r. L3 at 100 μg/mL.	Dube, Raphane et al., 2022 [117]
*Spondias mombin*	S.v.	EtOH extract and fractions.	In vitro	Larvicidal (L3) % activity. Fraction 4 at 400 μg/mL showed 100% mortality at 4 h. Also, at 50 μg/mL caused 100% mortality 12 h after exposure.	Medeiros et al., 2022 [118]
Compilation of many plants	*Strongyloides* spp. (S.p., S.r., S.v., and S.s.)	Plant extracts, fractions, and phytometabolites.	In vivo and In vitro	Bibliographic review of plants with activity against strongyloidosis.	Soleimani et al., 2022 [63]
*Artemisia brevifolia* and *N. tabacum*	*Strongyloides* spp., and S.p.	*A. brevifolia*: MeOH and Aq. extract. *N. tabacum*: Crude Aq. and MeOH extract.	In vivo and In vitro	*A. brevifolia*: In vivo inhibition of FEC assay against S.p. in sheep. *N. tabacum*: In vivo inhibition of FEC assay against S.p. in sheep.	Jato et al., 2022 [119]
*Zanthoxylum zanthoxyloides* and *Newbouldia laevis*	S.r.	EO of *Zanthoxylum zanthoxyloides* seeds and *Newbouldia laevis* leaves.	In vitro	FEC reduction.	Panda et al., 2022 [120]
*Plectranthus* *neochilus*	S.r.	EO	In vitro	Effective against S.r.	Raj & Kohli, 2022 [121]
*Siparuna guianensis*	S.v.	Hexane extracts of leaves.	In vitro	Significant inhibitory effect on the vitality of adult male worms	Ahmed et al., 2023 [52]
*A. negra*, *Momordica charantia*, and *Spondias mombin*	S.p. and *Strongyloides* spp.	*A. negra*: Tannin. *M. charantia*: Saponins, flavonoids, and anthocyannins. *S. mombin*: Aq. and EtOH crude extracts.	In vivo and In vitro	*A. negra*: Significantly lower FEC of S.p. and other gastrointestinal nematodes after 8 weeks. *Momordica charantia*: Showed efficacy against Strongyloides sp. in the cattle. *S. mombin*: Dose-dependent anthelmintic (reduction in LDI and FEC) activity.	Azeez Olanrewaju et al., 2023 [122]
Chemically synthesized natural compounds	S.p.	24 h exposure to 1% solutions of glutaraldehyde, thioacetic acid, 3-furoic acid, diethyl malonate, 2-oxopentanedioic acid, butan-1-amine, isovaleric acid, ethyl acetoacetate, phenol, and naphthol-2 compounds.	In vitro	The results of the study consider 28 compounds as promising for further research on anti-nematode activity. Compounds killed 100% of the S.p. larvae of all the development stages.	Boyko and Brygadyrenko, 2023 [123]
*Cucurbita moschata* and *C. papaya*	*Strongyloides* spp.	Squash and papaya seeds.	In vivo and In vitro	Anti-helminthic effect on gastrointestinal parasites in chicken (*Gallus gallus domesticus*).	Catedral et al., 2023 [124]
*Cucurbita pepo*	S.v.	Nps made by pumpkin seed oil loaded with IVM.	In vitro	IVM-loaded Nps demonstrated enhanced anthelmintic activity against S.v. in comparison to free IVM.	de Souza et al., 2023 [125]
A variety of Brazilian medicinal plants	*Strongyloides* sp., *Strongyloides* spp., S.s. and other nematodes	Diverse methods of preparation.	In vivo and In vitro	A review of diverse anthelmintic properties	Kuhn Agnes et al., 2023 [126]
Garlic	*Strongyloides* sp.	Administration of garlic in diet.	In vivo	Dose-dependent anthelmintic activity and FEC reduction and counts post-treatments from coprocultures of adult sheep. Increases in blood mass.	Parsaeimehr et al., 2023 [127]
*Turraea vogelii*, *Senna podocarpa*, and *Jaundea pinnata*	S.v.	Evaluation of the crude powder of the leaf extracts was extracted using different organic solvents *n*-hexane, ethyl acetate, and absolute methanol.	In vitro	In vitro activity against S.v. third-stage larvae.	Busari et al., 2024 [128]
*Ruta chalepensis*	S.v.	Crude MeOH extract and *n*-hexane, chloroform, and methanol partitions.	In vitro	In vitro activity against S.v. third-stage larvae.	Rodríguez-Garza et al., 2024 [12]
A variety of Mexican medicinal plants: *Amphipterygium adstringens*, *Artemisia ludoviciana*, *Cymbopogon citratus*, *Heterotheca inuloides*, *Jatropha dioica*, *Justicia spicigera*, *Larrea tridentata*, *Mimosa tenuiflora*, *Psacalium decompositum*, *R. chalepensis*, *Semialarium mexicanum*, and *Smilax aspera*	S.v.	Crude MeOH extracts.	In vitro	In vitro activity against S.v. third-stage larvae.	Rodríguez-Garza et al., 2024 [12]
*A. sativum* and *Artemisia absinthium*	S.rn	Alcoholic extracts (70% EtOH).	In vivo	The current study demonstrated that administering powdered of *A. sativum* bulbs and *A. absinthium* aerial parts at doses of 180 mg/kg/day and 90 mg/kg/day, respectively, for ten consecutive days, may be effective against digestive parasites in swine. The findings of the present study revealed that *A. sativum* and *A. absinthium* have the potential of treating gastrointestinal parasitoses in swine.	Băieş et al., 2024 [64]
*A. mexicana*, *J. dioica*, *Lippia graveolens*, *Thymus vulgaris*, and *Kalanchoe daigremontiana*	S.v.	Crude MeOH extracts.	In vitro	In vitro activity against S.v. third-stage larvae.	Elizondo-Luévano et al., 2025 [55]

%: Percent; Aq: Aqueous; EO: Essential oil; EtOAc: Ethyl acetate; EtOH: Ethanol; FEC: Fecal egg counts; S.p.: *Strongyloides papillosus*; S.rn: *Strongyloides ransomi*; S.r.: *Strongyloides ratti*; S.s.: *S. stercoralis*; S.v.: *Strongyloides venezuelensis*; S.w.: *Strongyloides westeri*; IVM: Ivermectin; MeOH: Methanol; Nps: Nanoparticles; IC_50_: 50% inhibition concentration; IC_90_: 90% inhibition concentration; LC_50_: 50% lethal concentration; SEM: Scanning electron microscopy; LM: Light microscopy; TEM: Transmission electron microscopy; LDI: Larval development inhibition; and LDT: Larval development test.

**Table 6:** Potential biomolecules with anti-*Strongyloides*’s activity.

Plant Species/ Biomolecule	*Strongyloides* Specie	Formulation	Model	Activity	Authors, Published Year [Reference]
Isolated isoquinoline alkaloids from *Macleaya cordata*, *Chelidonium majus*, *Corydalis turtschaninovii*, and *Corydalis Tuber*	S.r. and S.v.	Isolated isoquinoline alkaloids.	In vitro	PC_50_ in μM at 24 h: Against S.r.: D-Chelidonine = 11; Allocryptopine = 61; Protopine = 52; Berberine = 60; Dehydrocorydaline = 32; D-Corydaline = 18; L-Stylopine: 14; Emetine = 34; Papaverine = 54. Against S.v.: Chelerythrine = 72; Sanguinarine = 60; Allocryptopine = 51; Protopine = 33; Berberine = 32; Coptisine = 48; Dehydrocorydaline = 12; D-Corydaline = 30; L-Stylopine = 13; DL-Tetrahydropalmatine = 32; Emetine = 27; and Papaverine = 3.	Satou et al., 2002 [130]
Flavorings	S.rn.	Benzaldehyde, citral, D-limonene, and β-ionone.	In vitro	β-ionone and D-limonene at 10 g/L after 24 h caused the death of <50% of S.rn. larvae.	Boyko & Brygadyrenko, 2017 [131]
Secondary metabolites	S.s. and some STHs	A variety of secondary metabolites.	In vivo and In vitro	A variety of anthelmintic properties.	Romero-Benavides et al., 2017 [99]
Berberine from *A. mexicana*	S.v.	Purified alkaloid.	In vitro	LC_50_ in μg/mL at 96 h: Berberine = 1.6.	Elizondo-Luévano, et al., 2021a [109]
Berberine, curcumin, and quercetin	S.v.	Pure alkaloid, pure polyphenol, and pure flavonoid.	In vitro	IC_50_ in μM at 72 h: Berberine = 2; curcumin = 14; and quercetin = 111.	Elizondo-Luévano et al., 2020 [21]
Alkaloids	S.p. and against gastrointestinal nematodes	Berberine, harmaline, and piperine.	In vitro	Dose-dependent anthelmintic activity. The anthelmintic activity of alkaloids was evaluated using the egg hatch (EHA) and larval motility (LMA) assays.	da Silva et al., 2021 [132]
Products found naturally	S.r.	Aconitine (pseudoalkaloid), inositol (polyol), and selamectin (avermectin).	In vitro	Effect on L3 and adult worms (dead after 72 h), at 100 and 10 µM, respectively.	Keiser, J., & Häberli, C., 2021 [133]
Secondary metabolites from *Ozoroa insignis*	S.r.	Isolated compounds.	In vitro	Larvicidal (L3) % activity. Activity %: Anacardic acid 10 μM = 25 100 μM = 100 Ginkgolic acid 10 μM = 0 100 μM = 100 3-[7(Z)-pentadecenyl] phenol 10 μM = 25 100 μM = 100	Dube et al., 2021 [134]
Alkaloids	S.r. and S.v.	Isoquinoline alkaloids.	In vivo and In vitro	Isoquinoline alkaloids have shown strong wormicidal activity.	Nikelo et al., 2022 [135]
Eugenol, Isoeugenol, Thymol, and Carvacrol	S.p.	Essential oil.	In vitro	Eugenol, isoeugenol, thymol, and carvacrol are promising compounds against S.p. in L1, L2, and L3 stages.	Boyko, O., & Brygadyrenko, V., 2023 [136]
Oleic acid	S.p.	ω-9 monounsaturated fatty acid.	In vitro	23.5% mortality of nematode larvae in 1% solution.	Boyko, O., & Brygadyrenko, V., 2023 [123]
Secondary metabolites from *R. chalepensis*	S.v.	Coumarins and chalepin.	In vitro	Dose-dependent anthelmintic activity.	Rodríguez-Garza et al., 2024 [12]
2-aryl quinolines	S.r.	Different derivative compounds.	In vitro and In silico	Nematocidal activity against S.r. larvae.	Shanley et al., 2024 [137]
Chalepensin and Graveoline	S.v.	Chalepensin and Graveoline Isolated from *R. chalepensis*.	In vitro	Nematocidal activity against S.v. L3. LC_50_ in µM: Chalepensin 5.7 at 24 h 3.9 at 48 h 3.4 at 72 h Graveoline 28.3 at 24 h 25.9 at 48 h 24.4 at 72 h Nematocidal activity against S.v. parasitic female adults. LC_50_ in µM: Chalepensin 17.3 at 24 h 17.1 at 48 h 16.8 at 72 h Graveoline 27.8 at 24 h 26.9 at 48 h 26.5 at 72 h	Rodríguez-Garza et al., 2025 [54]

%: Percent; ω: Omega; S.p.: *Strongyloides papillosus*; S.rn: *Strongyloides ransomi*; S.r.: *Strongyloides ratti*; S.s.: *S. stercoralis*; S.v.: *Strongyloides venezuelensis*; S.w.: *Strongyloides westeri*; PC_50_: 50% paralysis concentration; IC_50_: 50% inhibition concentration; and LC_50_: 50% lethal concentration.

**Table 7:** Molecular targets and in silico binding of anti-*Strongyloides* phytocompounds.

Compound	Target Protein	Binding Site	Reference
Berberine	β-tubulin (human model)	Colchicine-binding site	[138]
Apigenin, kaempferol, and quercetin	β-tubulin (purified)	Colchicine site	[139,140]
Fisetin	β-tubulin	Paclitaxel site	[141]
Thymoquinone	α and β-tubulin heterodimer	Competes at colchicine site	[142]

## Data Availability

Not applicable.

## Abbreviations

The following abbreviations are used in this manuscript:

%	Percent
EO	Essential oil
FEC	Fecal egg counts
IVM	Ivermectin
MeOH	Methanol
NO	Nitric oxide
NPs	Nanoparticles
SEM	Scanning electron microscopy
TEM	Transmission electron microscopy
LM	Light microscopy
LDI	Larval development inhibition
LDT	Larval development test
PC_50_	50% paralysis concentration
IC_50_	50% inhibition concentration
IC_90_	90% inhibition concentration
LC_50_	50% lethal concentration
S.p.	*Strongyloides papillosus*
S.rn	*Strongyloides ransomi*
S.r.	*Strongyloides ratti*
S.s.	*Strongyloides stercoralis*
S.v.	*Strongyloides venezuelensis*
S.w.	*Strongyloides westeri*
STH	Soil-transmitted helminths
